# Self‐Healable Multifunctional Fibers via Thermal Drawing

**DOI:** 10.1002/advs.202400785

**Published:** 2024-04-29

**Authors:** Miao Qi, Yanting Liu, Zhe Wang, Shixing Yuan, Kaiwei Li, Qichong Zhang, Mengxiao Chen, Lei Wei

**Affiliations:** ^1^ College of Biomedical Engineering & Instrument Science Key Laboratory for Biomedical Engineering of Ministry of Education Zhejiang University Hangzhou 310027 China; ^2^ Zhejiang Lab Hangzhou 311100 China; ^3^ School of Electrical and Electronic Engineering Nanyang Technological University 50 Nanyang Avenue Singapore 639798 Singapore; ^4^ Key Laboratory of Bionic Engineering of Ministry of Education Jilin University Changchun 130022 China; ^5^ Suzhou Institute of Nano‐Tech and Nano‐Bionics Chinese Academy of Sciences Suzhou 215123 China

**Keywords:** characterization of self‐healable materials, self‐healable fibers, soft actuators, strain sensors, thermal drawing technique

## Abstract

The development of soft electronics and soft fiber devices has significantly advanced flexible and wearable technology. However, they still face the risk of damage when exposed to sharp objects in real‐life applications. Taking inspiration from nature, self‐healable materials that can restore their physical properties after external damage offer a solution to this problem. Nevertheless, large‐scale production of self‐healable fibers is currently constrained. To address this limitation, this study leverages the thermal drawing technique to create elastic and stretchable self‐healable thermoplastic polyurethane (STPU) fibers, enabling cost‐effective mass production of such functional fibers. Furthermore, despite substantial research into the mechanisms of self‐healable materials, quantifying their healing speed and time poses a persistent challenge. Thus, transmission spectra are employed as a monitoring tool to observe the real‐time self‐healing process, facilitating an in‐depth investigation into the healing kinetics and efficiency. The versatility of the fabricated self‐healable fiber extends to its ability to be doped with a wide range of functional materials, including dye molecules and magnetic microparticles, which enables modular assembly to develop distributed strain sensors and soft actuators. These achievements highlight the potential applications of self‐healable fibers that seamlessly integrate with daily lives and open up new possibilities in various industries.

## Graphical abstract

Mass production of self‐healable fibers is achieved via the thermal drawing technique. Real‐time monitoring of the self‐healing process is implemented by tracking changes in the transmission spectrum, allowing for precise determination of healing time and speed. Incorporating dye molecules and magnetic microparticles into the polymer matrix facilitates the creation of distributed strain sensors and soft actuators through modular assembly.

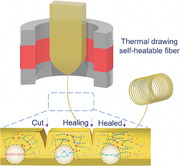

## 1 Introduction

Elastic and stretchable fiber devices have garnered much attention due to their high flexibility, elasticity, mechanical toughness, and ease of fabrication.^[^ ^1^, ^2^ ^]^ The vigorous development of elastic and stretchable fibers makes up for the shortcomings of traditional silica fibers that are fragile and limited in strain, especially when applied in wearable textiles and optomechanical sensing applications.^[^ ^3^, ^4^, ^5^, ^6^, ^7^, ^8^ ^]^ So far, these fibers have shown promising performance in strain sensors,^[^ ^9^, ^10^, ^11^ ^]^ temperature sensors,^[^ ^12^ ^]^ robotics and automation,^[^ ^13^, ^14^ ^]^ in vivo optogenetic modulations,^[^ ^15^, ^16^ ^]^ optoelectronic probes,^[^ ^17^, ^18^, ^19^ ^]^ and energy storage.^[^ ^20^, ^21^, ^22^, ^23^ ^]^ A variety of materials have been used for fabricating elastic and stretchable fibers, such as polydimethylsiloxane,^[^ ^24^ ^]^ hydrogels,^[^ ^25^, ^26^ ^]^ and biomaterials.^[^ ^27^, ^28^ ^]^ However, these thermoset elastomers typically require shaping into fibers before curing, as they become difficult to soften and reshape once crosslinked.^[^ ^29^, ^30^ ^]^ Furthermore, due to thermoset elastomers' inherent low mechanical strength, handling ultra‐thin fibers still poses challenges. Thermoplastic elastomers (TPEs) with melt‐processability offer a potential solution to overcome these limitations. TPEs are polymers that become soft and moldable above their glass transition temperature (*T*g), making them suitable for various polymer processing techniques.^[^ ^31^, ^32^, ^33^ ^]^ They are compatible with various processing techniques to produce long fibers. For example, the thermal drawing technique commonly used for glass fiber production involves heating a macroscopic preform, which is then drawn into a kilometer‐long microstructure fiber while preserving its geometry but reducing the cross‐sectional dimension.^[^ ^34^, ^35^ ^]^ To date, the thermal drawing technique has been exploited to produce TPE fibers and shown unique endowments, enabling the mass fabrication of multimaterial fibers with fine diameters and delicate structures.^[^ ^36^, ^37^, ^38^ ^]^

Another primary concern is the weakened properties of elastic and stretchable fibers when damaged. Inspired by nature, self‐healable materials can recover their physical properties after damage.^[^ ^39^ ^]^ Two main self‐healing mechanisms exist: extrinsic approaches to encapsulate healing agents and chemical approaches to incorporate supramolecular chemistry and dynamic bonds.^[^ ^40^, ^41^ ^]^ Encouragingly, the self‐healing property has been successfully introduced into TPEs through the dynamic exchange of disulfide bonds, metal coordination bonds, and hydrogen bonds, exploiting application scenarios for elastic and stretchable fibers.^[^ ^42^, ^43^ ^]^ Tan et al. fabricated covalently cross‐linked self‐healable ionogel fibers by melt‐spinning approach and innovatively demonstrated their application in flexible electronic devices.^[^ ^44^ ^]^ However, most self‐healable fibers are primarily fabricated by molding or spin‐coating, limiting their large‐scale manufacturing and application opportunities.^[^ ^45^, ^46^, ^47^, ^48^ ^]^ In addition, current characterization methods for self‐healable materials mainly fall into two categories: direct visual observation of the material morphology or indirect measurement of the functional integrity.^[^ ^49^, ^50^, ^51^ ^]^ There are significant differences between various test methods, so it is difficult to compare the healing effects of different self‐healable materials horizontally. Moreover, most current characterization methods are performed at certain points in the healing process and lack real‐time monitoring. Hence, there is an urgent need to establish a unified standard for quantifying the speed and time of self‐healing, especially for real‐time monitoring systems.^[^ ^52^ ^]^

This work employs the thermal drawing technique with mass production capability to manufacture self‐healable multifunctional fibers. Specifically, a self‐healable thermoplastic polyurethane (STPU) material is synthesized, containing dynamic reversible aromatic disulfide bonds, hard asymmetric alicyclic segments, and soft polytetramethylene ether glycol (PTMEG) segments. The self‐healing property of the synthesized material relies on the disulfide bond exchange reaction, while the hard segment domains enhance the mechanical performance. Subsequently, the synthesized material is molded into a cuboid‐shaped preform and then drawn into a microscopic functional fiber through the thermal drawing technique. Next, taking inspiration from commonly used optical fiber testing, the self‐healing process is quantitively studied by real‐time monitoring of the fiber transmission spectrum throughout the segment cutting, resplicing, and healing phases. Multifunctional capabilities are demonstrated: 1) distributed strain sensors are achieved by incorporating methylene blue and rhodamine 590 dye molecules, and 2) magnetic self‐healable soft actuators with four distinct modes of deformation and locomotion. These results highlight the potential applications of self‐healable fibers and unlock further opportunities across various fields in material characterization, fiber device fabrication, sensing, and soft robotics.

## 2 Results and Discussion

### 2.1 Fabrication of Self‐Healable Multifunctional Fiber via Thermal Drawing

The STPU material is synthesized through a two‐step reaction. To start, the prepolymer was obtained by step‐growth polymerization of thoroughly dried PTMEG and isophorone diisocyanate in DMAc at 70 °C. Subsequently, the mixture was cooled to 40 °C and further polymerized with bis(4‐hydroxyphenyl) disulfide. The detailed information can be found in Figures S1–S5 (Supporting Information). Due to dynamic disulfide bonds, the synthesized polymer exhibits disulfide metathesis and automatic repair capabilities when subjected to damage. In addition, incorporating loosely packed asymmetric alicyclic segments into soft PTMEG segments can effectively enhance mechanical performance while maintaining self‐healing properties.^[^ ^53^ ^]^

**Figure 1:**
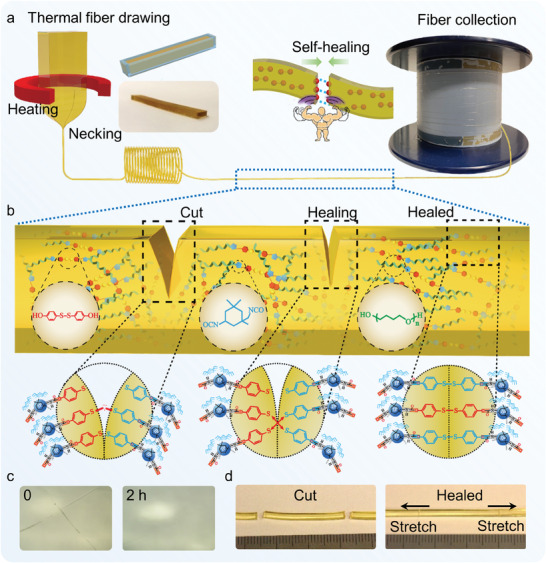
Fabrication of self‐healable fibers via preform‐to‐fiber thermal drawing technique. a) The as‐synthesized STPU material forms into a preform through a molding method. Then, the preform is fed into the tube furnace of the fiber drawing tower, where the bottom of the preform undergoes controlled softening and necking down under an externally applied force. Finally, the fiber is collected onto a cylindrical bobbin. b) Schematic diagram illustrating the self‐healing process after damage, wherein reversible metathesis of broken disulfide bonds facilitates the repair of cut materials. c) The surface of the self‐healable fiber healed in 2 h at room temperature against scratching. d) The self‐healable fiber exhibited outstanding strength against external forces after the instant healing.

Then, in order to construct a preform applicable to the fiber thermal drawing process, a DMAc solution with synthetic STPU was poured into a designed cuboid‐shaped PTFE mold after the synthetic procedure (**Figure** **1**a). Then, the preform material was subjected to continuous heating, gradually increasing the temperature from 60 to 130 °C throughout 48 h, to facilitate evaporation of DMAc. A well‐shaped preform was obtained after being baked in a vacuum oven at 130 °C for 24 h.

Next, the prepared preform was loaded into a two‐zone vertical tube furnace on a fiber drawing tower (Figure 1a). During the thermal drawing process, the bottom portion of the preform was softened by the prescribed heating temperature and underwent necking by the applied external force. Subsequently, the preform was severed from the necking region, and its end was elongated to affix onto a pair of rotating rollers below for drawing. The material's viscosity ensures that the fiber's cross‐sectional shape aligns consistently with the preform. The viscosity property of the STPU was determined using a rheometer (TA HR10) under steady shear and dynamic oscillatory conditions, with in situ heating from 90 to 150 °C. Figure S6 (Supporting Information) shows the complex shear viscosity (η*), the storage (G′), and the loss (G′′) modulus. Both G′ and G″, as well as |η*|, decrease as temperature rises. A transition point is observed where G′ and G″ curves intersect. Below this point, elastic behavior dominates; above it, flow is favored by G″ prevailing. This crossover point satisfies the rheological requirements for thermal drawing, making the synthesized STPU suitable for processing through this method. The preform was fed down into the furnace at a speed of 1 mm min^−1^, while the fiber was drawn at a speed of 0.4 m min^−1^ by the rotating rollers. Finally, the self‐healable fiber with a cross‐sectional dimension of 1 mm × 0.5 mm was collected by a cylindrical bobbin.

**Figure 2:**
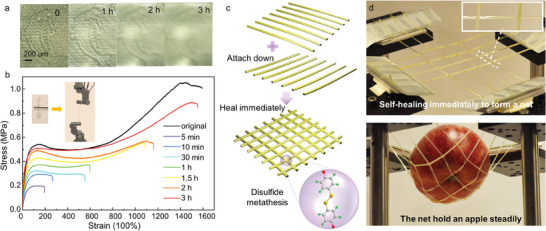
Self‐healing performances of the fabricated STPU fibers and their surface. a) Optical microscopy images of dolphin‐patterned STPU surface during the healing process. b) Stress–strain curves of the original STPU fiber and respliced fibers after different healing times. Inset: Photograph of STPU fiber attached to cardboard during stretching test. c) Schematic diagram of the formation process of a self‐healable net. The cut STPU fibers are divided into two groups and vertically stacked in a parallel arrangement, resulting in immediate net formation through disulfide bond metathesis. d) Photograph of STPU fibers self‐healed to form a net. The net can lift a 50 g apple steadily.

The fabricated fiber exhibits superior self‐healing properties at room temperature, owing to the disulfide metathesis, as shown in Figure 1b. To study the self‐healing performance of the fabricated TPU fibers, the surface of the self‐healable fiber was scratched and healed in 2 h at room temperature. Optical microscope images of the STPU surface scratched by the tip of a tweezer also show that the X‐shaped scratch gradually shallows with time and almost disappears after 2 h (Figure 1c). Moreover, a routine cut and healed method was conducted to confirm the self‐healing capability of the fabricated fiber. As illustrated in Figure 1d, self‐healable fiber was divided into three sections, respliced, and immediately stretched from both ends. Although complete healing of the fiber surface requires more time, the fiber was partially healed and was strong enough to resist external forces. Additionally, a dolphin stamp was printed on the STPU film. The disappearing process of the pattern was vividly displayed under microscopic observation, further revealing the self‐healing ability of the synthetic material (**Figure** **2**a).

The mechanical strength of the fabricated STPU fiber was measured by a uniaxial tensile test. Specifically, an STPU fiber with a cross‐sectional dimension of 0.5 mm × 0.3 mm was affixed to cardboard using glue at both ends, leaving a hanging portion of 5 mm in length. Subsequently, the sample was subjected to axial tension using a testing machine equipped with a 10 N load cell, applying a constant speed of 5 mm min^−1^ (the inset of Figure 2b). As depicted in Figure 2b, the mechanical behavior of the pristine STPU fiber aligns more closely with the stress‐strain model observed in softened glassy polymers,^[^ ^54^ ^]^ albeit exhibiting greater strain capacity. Following the yield point, necking occurs, and stress rises significantly during the latter half of the curve until the ultimate failure is reached. The fracture strength of the STPU fiber is measured to be 1.05 MPa, slightly lower than the traditional thermally drawn fibers previously reported.^[^ ^17^, ^36^, ^37^ ^]^ One possible explanation is the low molecular weight of TPU. For example, the molecular weights of commercial TPEs are generally hundreds of thousands or even higher, while the molecular weight of our synthesized TPU is only ≈40 000. Notably, this STPU fiber demonstrates exceptional stretchability, with an elongation rate as high as 1600%. To investigate its self‐healing performance, multiple samples were prepared by attaching STPU fibers onto cardboard, cutting them in half, and realigning them. Tensile tests were carried out under the same conditions after different healing periods at room temperature. The fracture stress and strain of STPU fibers increase with the extension of healing time, as illustrated in Figure 2b. After a healing period of 3 h at room temperature, the STPU fiber can withstand a fracture stress of 0.89 MPa and reach a fracture strain of 1550%. This indicates that the mechanical property of the STPU fiber is almost restored through self‐healing within a relatively short duration. The fiber was cut and spliced into a net configuration to further validate its mechanical strength and self‐healing capability for practical applications (Figure 2c). Due to the dynamic reaction of the disulfide bonds, immediate self‐healing occurred at the junctions upon connection. Subsequently, a 50 g apple was placed on the net, demonstrating its stable supporting capability for real‐life scenarios (Figure 2d).

### 2.2 Real‐Time Monitoring Transmission Spectra of the Self‐Healing Process

**Figure 3:**
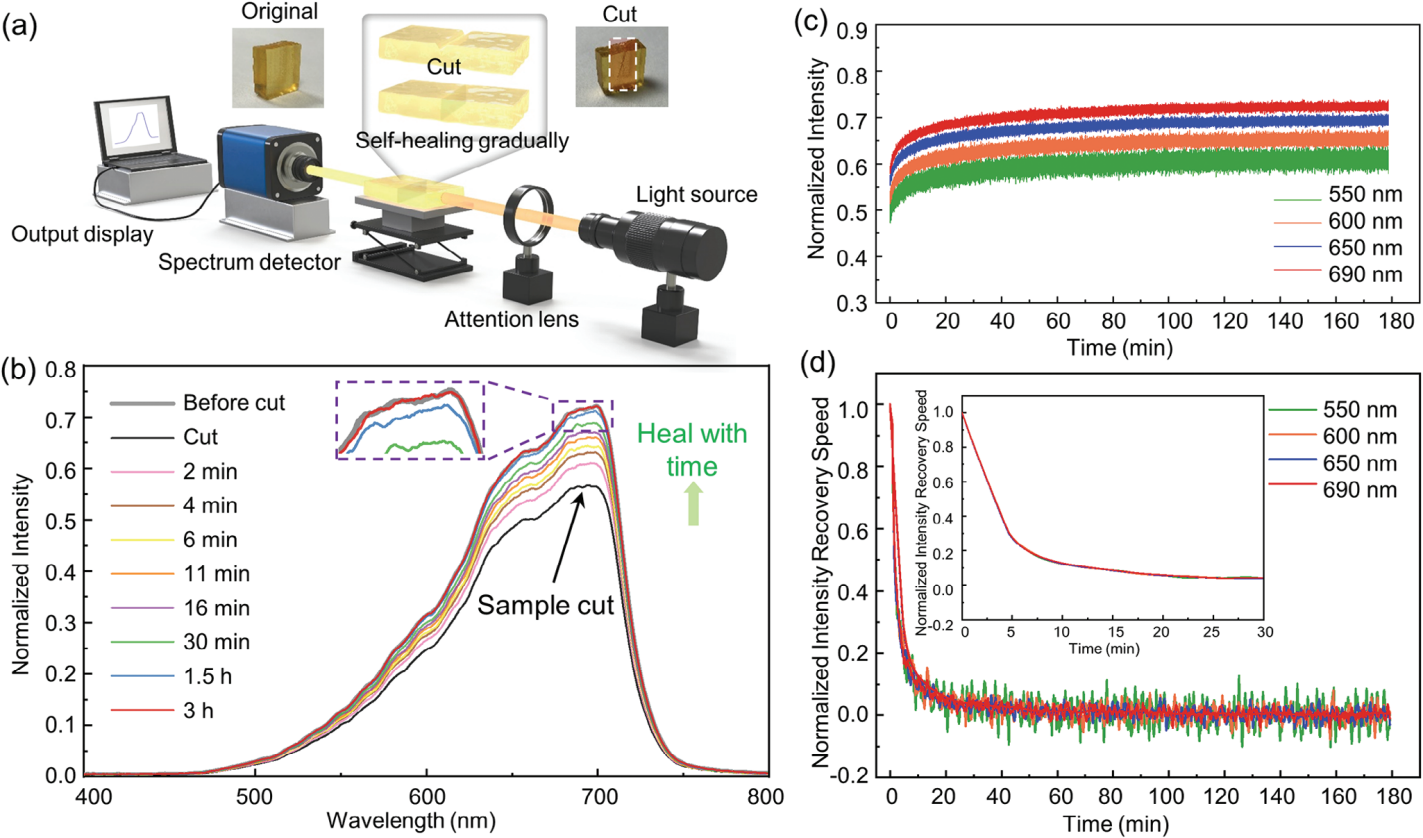
Direct monitoring of the self‐healing process in real‐time. a) Schematic diagram of the spectrometer setup for monitoring the self‐healing process. b) Transmission spectra recovery along the STPU block self‐healing process. c) The transmission spectra at 550, 600, 650, and 690 nm over time in the self‐healing process. d) The differentiation of the transmission spectra reveals the recovery speed during the self‐healing process: it heals the fastest at the beginning, and the healing speed decreases with time. Inset: The smoothed recovery speed within 30 min.

In addition to the conventional indirect characterization of the self‐healing performance, an optical approach is proposed for quantitatively characterizing the time and speed of self‐healing. Specifically, an STPU sample was cut into two pieces and placed in the optical path between a white light source (Fiber‐Lite DC950 Illuminator) and a miniature spectrometer (Ocean Optics USB2000+) (**Figure** **3**a). Real‐time monitoring of the variation in transmitted light during the self‐healing process was performed. As depicted in Figure 3b, the black curve represents the transmission spectrum after dividing the sample into two sections. Compared to the pre‐cut spectrum (the gray curve), the normalized transmission intensity experiences a decrease due to the diffuse reflection caused by the roughness at the cut cross‐section. Since no absorption peak exists for STPU within the 550–750 nm range (Figure S5, Supporting Information), variations in spectral decline at different wavelengths correspond to changes in relative spectral radiance from the light source,^[^ ^55^ ^]^ with maximum change observed at 699 nm where the intensity is the highest, decreasing from 0.72 to 0.56. With the gradual self‐healing of the cut cross‐section, the transmission spectrum gradually recovers. After 6 min, half of the reduced transmission intensity is restored (the blue curve in Figure 3b). The recovery speed gradually slows, and after 3 h, the transmission spectrum almost entirely coincides with the pre‐cut curve, indicating that the STPU sample is completely self‐healed. The real‐time variations in transmission intensity at different wavelengths were recorded to further investigate the self‐healing process. As presented in Figure 3c, four representative wavelengths are selected. Taking 550 nm as an example, the transmission intensity increases rapidly in the first few minutes and then gradually slows down. The noise in the spectrum decreases with longer wavelengths due to the increased relative spectral radiance of the incident light at the corresponding wavelength. Obviously, the transmission spectra at different wavelengths exhibit identical varying tendencies. Self‐healing occurs instantly after re‐splicing the STPU and slows down until complete healing is achieved. This self‐healing process elucidates why the fiber can immediately withstand external tension upon reconnection. As shown in Figure 1c, partial healing occurs internally despite surface cracks still being present. Next, derivative analysis is performed on the transmission spectra to determine recovery speeds during the self‐healing process. As shown in Figure 3d, all spectra exhibit maximum recovery speeds at the beginning, dramatically decreasing over time until stabilizing at relatively low values after 30 min. The observed trend of self‐healing speed obtained from transmission spectra aligns with previously reported disulfide displacement reaction rates found in literature since self‐healing relies on disulfide metathesis.^[^ ^56^ ^]^

The method of recording the transmission spectrum variation to characterize the self‐healing process of materials exhibits notable merits and promising prospects. First, this method provides a standardized measurement to assess the light‐permeable material's self‐healing speed and recovery extent as the spectral change is visualized and quantifiable. Secondly, unlike other methods that select several points during the self‐healing process for testing, this approach enables real‐time and coherent observation. Thirdly, the approach is simple, only requiring self‐healable materials to be placed along the optical path. Finally, this method can theoretically be applied to all light‐permeable materials. Encouragingly, most self‐healable materials are transparent, including hydrogels, biomaterials, and self‐healable TPEs. In addition, light sources and spectrometers can be replaced with other wavelength ranges, such as ultraviolet, infrared, etc., further expanding the range of characterizable materials. Therefore, this method has the potential to be applied to a broader range of self‐healable materials.

### 2.3 Distributed Strain Sensor Based on Self‐Healable Fibers

As the spectral changes in these fibers can accurately reflect the strains induced by human motion, elastic and stretchable optical fibers have gained significant attention. As discussed, STPU fibers exhibit a superior stretchability of 1600% strain, making them a promising candidate for strain‐sensing applications. In addition, the self‐healing property of STPU enables the modular assembly of different STPU fibers with distinct absorption peaks into a distributed strain sensor configuration. To demonstrate the feasibility of this approach, two dye molecules with varying absorption spectra were separately incorporated into synthetic STPU solutions before molding. These solutions were then poured into rectangular‐shaped molds and subjected to vacuum heating for DMAc solvent evaporation. Figure S7 (Supporting Information) illustrates three types of resulting STPU films: original film without doping, rhodamine 590‐doped film, and methylene blue‐doped film, each having a thickness of 120 µm. The black line represents the absorption spectrum of the undoped STPU film, exhibiting no prominent absorption peak. In contrast, rhodamine 590‐doped and methylene blue‐doped STPU films exhibit absorption peaks at wavelengths of 533 and 631 nm, respectively. Next, rhodamine 590 and methylene blue‐doped STPU fibers were fabricated. The transmission spectrum of the original STPU fiber is described by the green line in Figure S9 (Supporting Information). Notably, compared to the original fiber's transmission spectrum, the transmission spectrum of rhodamine 590‐doped fiber shows a significant drop within the wavelength range from 500 to 600 nm (orange line), indicating light absorption within this range. Similarly observed for methylene blue‐doped fiber (blue line), its transmission spectrum decreases within a wavelength range from 550 to 660 nm. The broad wavelength drop range is reasonable because the light propagates throughout the entire length of the fiber. Figure S10 (Supporting Information) displays the normalized absorption spectra extracted from the transmission spectra, corresponding to the absorption spectra obtained from UV‐vis tests.

**Figure 4:**
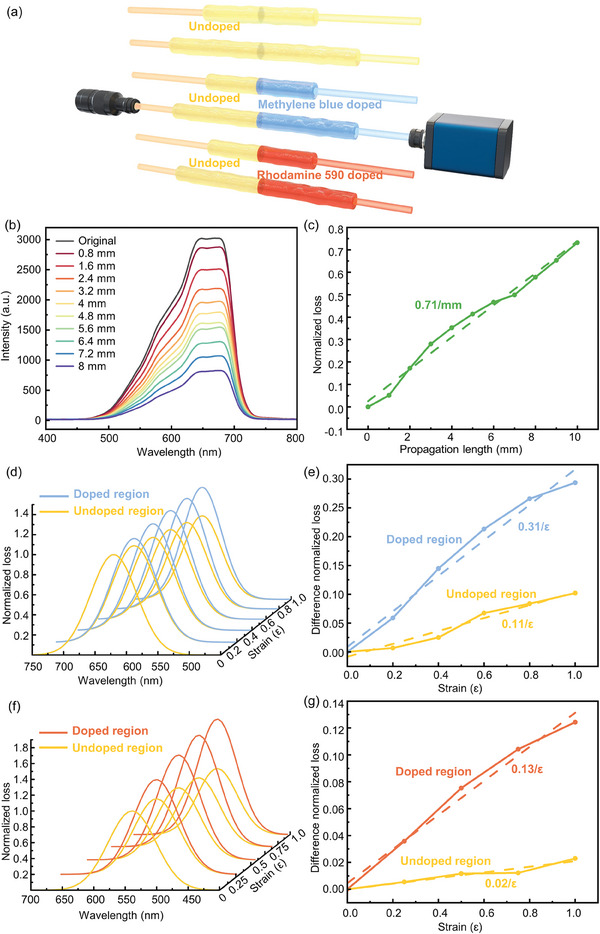
Methylene blue and rhodamine 590 doped STPU fiber for distributed strain sensing. a) Schematic of the testing approach. b) Transmission spectra variation of STPU fiber during stretching. c) The nearly linear increase of the normalized loss against propagation length. d) Normalized absorption spectra variation of the fabricated distributed strain sensor when the strain is applied to the undoped and methylene blue‐doped sensor regions. e) The nearly linear increase of difference loss against the applied strain. f) Normalized absorption spectra variation of the fabricated distributed strain sensor when the strain is applied to the undoped region and rhodamine 590 doped sensor region. g) A nearly linear increase in difference loss with respect to the applied strain.

Initially, the transmission spectrum alterations in the original STPU fiber during stretching were documented. Subsequently, a comparative analysis was conducted to examine the spectral changes in the dye molecular‐doped fibers under similar stretching conditions (**Figure** **4**a). Figure S8 (Supporting Information) shows the experimental setup where the STPU fiber is placed between a light source and a spectrometer while being fixed on two moveable stages for left and right stretching. The transmission spectra variation during stretching is shown in Figure 4b, indicating that the absorption of the fiber increases with the change in fiber length. The normalized loss of the STPU fiber exhibits a linear relationship with the propagation length (Figure 4c), indicating its suitability as a strain sensor. Subsequently, an original STPU fiber was spliced together with a methylene blue doped STPU fiber, followed by healing at room temperature for 3 h to fabricate a distributed strain sensor featuring a 4 mm dye‐doped region (Figure S11a, Supporting Information). This strain sensor is realized by recording the changes in the transmission spectra during the application of strain. The strain sensor was affixed onto a translation platform to perform stretching experiments, and the left displacement table was adjusted to stretch the undoped region. Each increment of stretching is set at 0.8 mm until reaching 8 mm after five repetitions. Subsequently, the right displacement table was adjusted to stretch the methylene blue‐doped area to 8 mm (Figure S11a, Supporting Information). As shown in Figure 4d, stretching the methylene blue‐doped region leads to a more pronounced increase in the normalized loss than stretching the undoped area (Figure S12, Supporting Information). Taking the absorption at 631 nm as the sensing indicator, the sensor provides a nearly linear response within a strain range of 100%. The difference in the normalized loss for the undoped region is 0.11/ε, whereas, for the methylene blue‐doped region, it reaches 0.31/ε (Figure 4e). This preliminary demonstration showcases a trustworthy distributed strain sensor that utilizes the self‐healing characteristic of STPU fiber. Similarly, an original STPU fiber and a rhodamine 590 doped STPU fiber were spliced together for strain sensing application (Figure S11b, Supporting Information). When the doped region is stretched to 1× strain, it results in twice the normalized loss compared to the undoped region (Figure 4f; Figure S13, Supporting Information). Taking the absorption at 533 nm as the sensing indicator, the difference normalized loss is 0.13/ε and 0.02/ε, respectively (Figure 4g).

### 2.4 Magnetic STPU Soft Actuators

**Figure 5:**
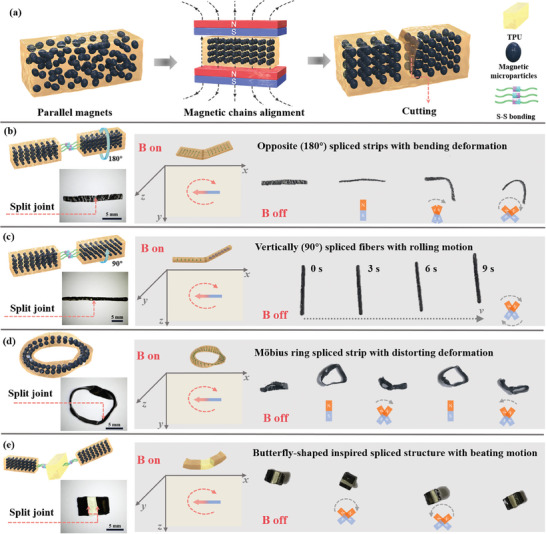
Magnetic STPU soft actuators. a) Schematic of the magnetic chain alignment in the TPU elastomer under the magnetic field. b) Opposite magnetization direction (180°) spliced strips‐based soft actuator with bending deformation under the *x–y* plane magnetic field. c) Vertically magnetization direction (90°) spliced fiber‐based soft actuator with the rolling motion under the *x–z* plane rotating magnetic field. d) Möbius ring spliced strip‐based soft actuator with distorting deformation under the *x–y* plane magnetic field. e) Butterfly‐shaped inspired spliced soft actuator under an *x–z* plane oscillating magnetic field.

In addition to dye molecules, magnetic particles can be incorporated into STPU to achieve multifunctional expansion. Compared with stiff materials, STPU, as an exemplary elastomer, possesses a low elastic modulus and is accessible to dope, making it a compelling candidate for soft actuators. For magnetic‐driven soft actuators, magnetic particles are embedded into the polymer matrix to fabricate magnetically responsive soft materials.^[^ ^57^ ^]^ Moreover, the magnetic particles‐embedded soft actuator exhibits unique and desirable features, including rapid response and remote actuation under a magnetic field.^[^ ^58^, ^59^ ^]^ In this work, to achieve various shape deformations and enable locomotion of the STPU elastomer‐based magnetic soft actuators, oriented magnetic microparticles are incorporated into the STPU polymer matrix. Initially, the magnetic microparticles are distributed randomly in the polymer matrix after mixing. Subsequently, they tend to change from a disordered arrangement to align along the direction of the applied magnetic field, as illustrated in **Figure** **5**a. Upon solidification, the oriented magnetized STPU elastomer is procured, wherein the soft polymer matrix has successfully immobilized the linearly aligned iron microparticles. Throughout our experiment, the magnetization direction of the magnetic microparticles is intentionally chosen to coincide with the horizontal plane of the STPU elastomer specimen.

Leveraging the inherent self‐healing properties of TPU, soft magnetic actuators with various geometrical configurations were constructed by splicing different modules together via the disulfide bonds. Ordinarily, the self‐healable magnetic soft actuator will endure magnetic torque until the oriented magnetic microparticles in the polymer matrix align with the direction of the applied external magnetic field (Figure S14, Supporting Information). Ordinarily, the self‐healable magnetic soft actuator will endure magnetic torque until the oriented magnetic microparticles in the polymer matrix align with the direction of the applied external magnetic field. Here, four types of magnetically responsive soft actuators are presented: bending, rolling, distorting, and beating. For the first type, Figure 5b illustrates a magnetic soft actuator exhibiting bending deformation. By bisecting STPU strips laden with the oriented magnetic microparticles inverting the right segment and then adhesively splice joining the left and right sections through self‐healing, opposite magnetization directions (180°) spliced STPU strips based soft actuator is successfully obtained. When subjected to an external magnetic field, this horizontally resting soft actuator first erects itself, then bends and recovers when the magnetic field rotates at an angle in the *x–y* plane, as demonstrated in Movie S1 (Supporting Information). Such bending deformation is caused by magnetic torque under the magnetic field, owing to the opposite magnetization directions within the soft actuator strip structure. For the second type, when an oriented magnetic STPU fiber with a circular cross‐section is bisected and the right half rotated by 90° before reassembling via the self‐healing process, a vertically spliced fiber‐based soft actuator is obtained. This type of self‐healable soft actuator achieves a rolling motion under the 360° rotating magnetic field in the *x–z* plane and moves forward along *x* direction as depicted in Figure 5c. The soft robot rolls forward owing to the pulling force and the torque under the rotating and straightforward magnetic field (Movie S2, Supporting Information). For the third type, the Möbius ring, a captivating structure, is constructed by twisting the oriented magnetic STPU strip and then splicing the two ends together. The magnetic Möbius ring stands upright when the magnetic field is off but transitions to rest flat, beginning its distorting deformation when the magnetic field rotates at an angle in the *x–y* plane, as shown in Figure 5d. The orientation distribution of magnetic microparticles in the strip alters due to the twist of the 2D circular structure, enabling the Möbius ring spliced strip soft actuator to exhibit distortion and folding deformations (Movie S3, Supporting Information). For the fourth type, a butterfly‐shaped inspired structure is constructed. Two rectangular magnetic STPU blocks are spliced into assembly with the pristine STPU block in the middle to form a butterfly‐shaped soft actuator by the self‐healing process. This soft actuator achieves the beating motion under an oscillating magnetic field in the *x–z* plane, reminiscent of a butterfly flapping its wings, as represented in Figure 5e and Movie S4 (Supporting Information). The controllable deformation and locomotion of these magnetic STPU‐based soft actuators demonstrate the immense potential to serve as integral components in soft robotics applications, including artificial muscles, autonomous soft robots, and soft grippers.

## 3 Conclusion

A stretchable thermoplastic elastomer TPU was synthesized via a step‐growth polymerization reaction, and the introduction of disulfide bonds endowed it with superior self‐healing properties. The synthesized TPU was employed in the thermal drawing process to fabricate a STPU fiber, thereby enabling the mass production of multifunctional self‐healing fibers. Furthermore, real‐time monitoring of the transmission spectrum variation of the STPU material during the self‐healing process enabled investigation into its healing speed, thereby establishing a standardized approach for monitoring and characterizing self‐healable materials. Additionally, STPU fibers with diverse peak absorption were fabricated by doping dye molecules, offering promising applications in distributed strain sensors. Finally, leveraging the inherent self‐healing capabilities of TPU allowed for the construction of soft magnetic actuators with various geometrical configurations to facilitate versatile motions. This work is expected to advance efforts in the processing, characterizing, and functionalizing of self‐healable materials. Also, by incorporating self‐healing attributes, this study is anticipated to prolong the operational lifespan of soft fiber devices and broaden their applications in diverse fields such as sensing and soft robotics.

## 4 Experimental Section

### Materials and methods

PTMEG, *N,N*‐dimethylacetamide (DMAc, anhydrous, 99.8%), dibutyltin dilaurate (DBTDL, 95%), and methylene blue were purchased from Sigma‐Aldrich. Isophorone diisocyanate (99%) and bis(4‐hydroxyphenyl) disulfide (98%) were purchased from Tokyo Chemical Industry. Rhodamine 590 was purchased from Luxottica Exciton. Iron powder (spherical, APS 6–10 microns, reduced, 99.5%) was purchased from Alfa Aesar. FTIR spectrum was obtained by a Nicolet iS10 spectrometer (Nicolet) with TPU solution coating on a KBr pellet. The ^1^H NMR spectrum was acquired by a JEOL ECA400 spectrometer (400 MHz) with CDCl_3_ as solvent. The molecular weight and molecular weight distribution were measured by gel permeation chromatography (GPC) (TRSEC MODLE302/PL220) using polystyrene calibration standards with THF as an elution solvent. The absorption spectra of STPU films were measured using a UV‐vis Spectrophotometer (Hewlett Packard 8453). The optical sensing system was tested with Ocean 2000 and a white source. The optical micrographs were obtained by Olympus BX51. Mechanical tensile‐stress experiments were performed by SAAS EUT4103X electronic universal testing machine.

### TPU synthesis

A 14.5 g of PTMEG (14.50 mmol) was added to a three‐necked, round‐bottomed flask and mechanically stirred at 100 °C for 1 h under vacuum to remove moisture. After being cooled to 70 °C, 6.77 g of isophorone diisocyanate (30.45 mmol) and 0.050 g of DBTDL (2000 ppm) dissolved in 5 mL DMAc was added dropwise into the flask. The mixture was purged with argon and stirred for 2 h to get the prepolymer. Next, the system was cooled to room temperature, and 10 mL of DMAc solution of 3.63 g of bis(4‐hydroxyphenyl) disulfide (14.50 mmol) was added. The mixture was heated to 40 °C and allowed to react for 1.5 h. The final concentration of TPU was adjusted to 30 wt.% by adding 43 mL of DMAc for next‐step characterization. FTIR (KBr): 3319 cm^−1^ (‐NH), 2267 cm^−1^ (N═C═O), 1653 cm^−1^ (O═C─NH), 1716 cm^−1^ (C═O). ^1^H NMR (400 MHz, CDCl_3_, δ): 7.39–7.22 (‐S‐S‐CCH, 4H), 6.92–6.70 (‐COOCCH, 4H), 4.18‐3.30 (─O─CH_2_, 56H), 3.05–2.76 (─NH─C**H**_2_, 2H), 1.88–1.49 (─O─CH_2─_C**H**_2_, 58H), 1.21–0.70 (─CCH_3_, 18H). THF‐GPC: *M_n_* = 26 400 g mol^−1^, *M_w_* = 39 900 g mol^−1^, PDI = 1.51.

### Preparation of STPU Preform and STPU Film

The STPU solution in DMAc with a concentration of 30 wt.% was poured into a rectangular‐shaped PTFE mold with dimensions of 150 mm × 20 mm × 20 mm. Subsequently, the mold was gradually heated from 60 to 80 °C on a hot plate over 48 h. Afterward, it was transferred to a vacuum oven and subjected to heating at 130 °C for an additional duration of 24 h to remove residual solvent. The vacuum oven was slowly inflated to keep the surface of the preform flat. Following this process, the preform was extracted and ready for thermal fiber drawing. STPU film was fabricated in a PTFE mold of 150 mm × 100 mm × 15 mm through the same process.

### STPU Fiber Fabrication via Thermal Drawing Technique

The prepared STPU preform was loaded into a two‐zone vertical tube furnace on a fiber drawing tower. The top‐zone temperature was set at 150 °C, and the bottom‐zone temperature was set at 250 °C. Following an incubation period, the lower section of the STPU preform underwent softening and necking under external force application. Subsequently, the necked region of the STPU preform was cut off, leaving behind a connected portion that was stretched into a thin strand. Then, the preform was fed down into the furnace at a constant speed of 1 mm min^−1^ while the fiber was drawn at a speed of 0.4 m min^−1^. The STPU fiber was collected on a cylindrical bobbin for further characterization and tests.

### Self‐Healing and Tensile Tests for STPU Fiber

The self‐healing performance of the STPU fiber was evaluated by a cut recovery experiment, in which an STPU fiber was divided into three portions and respliced. Additionally, scratch recovery tests were conducted on the STPU surface to visually observe the self‐healing process at room temperature using an optical microscope. The mechanical property of STPU fiber with a dimension of 5 mm × 0.5 mm × 0.3 mm was examined via an electromechanical universal test system with a 10 N load cell and a constant speed of 5 mm min^−1^. First, the two ends of the STPU fiber were fixed to the C‐shaped cardboard with glue, leaving a hanging part 5 mm long. Then, the suspension part was placed on the gasket so that it was flush with the cardboard at both ends. This effectively prevents the fibers from being stretched and twisted during the cutting process. After cutting, the two parts were reassembled by tweezers under a microscope. After fixing the two ends of the C‐shaped cardboard with fixtures, the cardboard was cut in the middle to conduct stress–strain testing.

### Fabrication of the Distributed Strain Sensors

The distributed strain sensors were prepared by doping organic dyes into TPU solution after synthesis. In brief, the obtained DMAc solution of TPU was divided into two equal parts and 10 mL of DMAc solutions (0.5 mm) of rhodamine 590 and methylene blue were separately added to each part. Following the same molding preform preparation and thermal fiber drawing process, two categories of STPU fibers with distinct absorption peaks were fabricated. An 8 mm long section from each type of fiber was cut, and these different sections were subsequently respliced together to obtain strain sensors with two distributed color regions under the self‐healing effect.

### Fabrication of the Magnetic‐Based STPU Elastomer

Iron microparticles were selected as the magnetic response elements to be embedded within the STPU polymer matrix. Following the two‐step polymerization reaction, magnetic microparticles were added and stirred vigorously with TPU solution. Subsequently, the mixture was poured and cast into the mold. Considering the balance between the self‐healing mechanical properties and magnetic properties of the two composites, the mass ratio of Fe to TPU was 1:20. Then, the mixture in the mold was then placed between the parallel magnet bars during the evaporation of the solvent and degassing process in the oven. An external magnetic field was applied to induce the formation of orientation and magnetization of iron microparticles in the STPU polymer matrix.

## Conflict of Interest

The authors declare no conflict of interest.

## Supporting information

Supporting Information

Supplemental Movie 1

Supplemental Movie 2

Supplemental Movie 3

Supplemental Movie 4

## Data Availability

The data that support the findings of this study are available from the corresponding author upon reasonable request.
