# Supplementary material for: Self‐Healable Multifunctional Fibers via Thermal Drawing
Source: Adv Sci (Weinh). 2024 Apr 29;11(24):2400785. doi: 10.1002/advs.202400785 (PMC11200011; doi:10.1002/advs.202400785)
Supplement: Supplementary file 1 — Supporting Information [file ADVS-11-2400785-s002.pdf]

ADVANCED SCIENCE
Open Access

Supporting Information

for *Adv. Sci.*, DOI 10.1002/advs.202400785

Self-Healable Multifunctional Fibers via Thermal Drawing

Miao Qi, Yanting Liu, Zhe Wang, Shixing Yuan, Kaiwei Li, Qichong Zhang, Mengxiao Chen and Lei Wei**

Supporting Information

Self-healable Multifunctional Fibers via Thermal Drawing

Miao Qi, Yanting Liu, Zhe Wang, Shixing Yuan, Kaiwei Li, Qichong Zhang, Mengxiao Chen, Lei Wei**

Synthesis of self-healable TPU: The self-healable thermoplastic polyurethane material is synthesized through a two-step reaction. Firstly, the prepolymer was obtained by step-growth polymerization of thoroughly dried PTMEG and isophorone diisocyanate in DMAc at 70 ^{o}C. Subsequently, the mixture was cooled to 40 ^{o}C and further polymerized with bis(4-hydroxyphenyl) disulfide.

FTIR characterization: For FTIR characterization, the synthesized TPU was diluted with DMAc and coated on a KBr pallet. Figure S2 illustrates that the absorption peaks at 2267 cm^{-1} and 1716 cm^{-1} correspond to N=C=O and C=O stretching bonds in DMAc, respectively. The absorption peaks at 1653 cm^{-1} and 3319 cm^{-1}, which are attributed to amide bonds and attached secondary amines, indicate the polymerization of hydroxyl groups with isocyanate groups. This confirms the chemical characteristics of the synthesized TPU.

^{1}H NMR characterization: The structural characterization of the synthesized TPU was verified by conducting a ^{1}H NMR spectrum (Figure S3). The signals corresponding to aromatic units appear at 7.39-6.70 ppm, the peaks of PTMEG protons appear at 4.18-3.30 and 1.88-1.49 ppm, while the peaks of methyl groups from isophorone diisocyanate appear at 1.21-0.70 ppm. The integral ratio of each peak area in the NMR spectrum is completely consistent with the molar ratio of the reagents added, further confirming successful synthesis.

GPC analysis: GPC analysis in the THF mobile phase provided molecular weight and distribution information for the synthesized TPU. A single peak corresponding to TPU appeared at around 19 min., indicating a narrow molecular weight distribution. Calculated by integration, the weight-average molecular weight of TPU is 39900 g/mol, the number-average molecular weight is 26400 g/mol, and the polydispersity index (PDI) is 1.51. It is worth mentioning that the scattered light intensity measured by GPC is proportional to the size cubed; thus higher molecular weights result in stronger signals. In short, the relatively large molecular weight and

narrow molecular weight distribution ensure that the synthesized TPU possesses sufficient mechanical strength.

UV-vis spectroscopy characterization: UV-vis spectroscopy was adopted to characterize the transmittance of the synthesized material. A thin film with a thickness of 120 μm was hot-pressed from the TPU for the transmittance test. As shown in Figure S5, the TPU exhibits good transparency and no obvious absorption peak within the visible light range, making it suitable for further optical characterizations and strain sensor applications. However, the transmittance of the TPU film is slightly lower than previously reported.[1] This can be attributed to surface roughness caused by the hot pressing and cooling process, leading to light scattering and reduced transmittance.

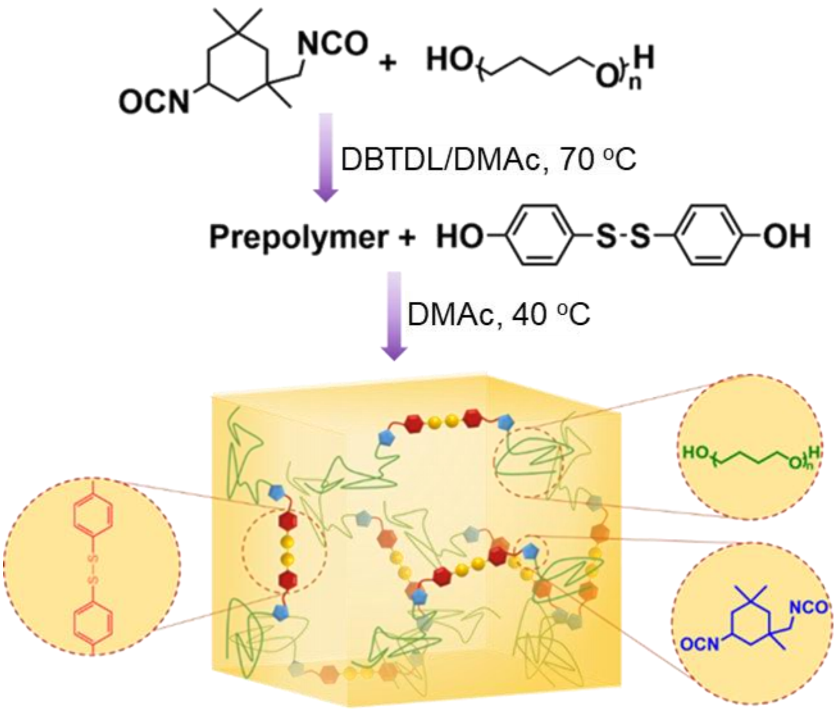

Figure S1. Synthesis route and molecular structure of the self-healable TPU.

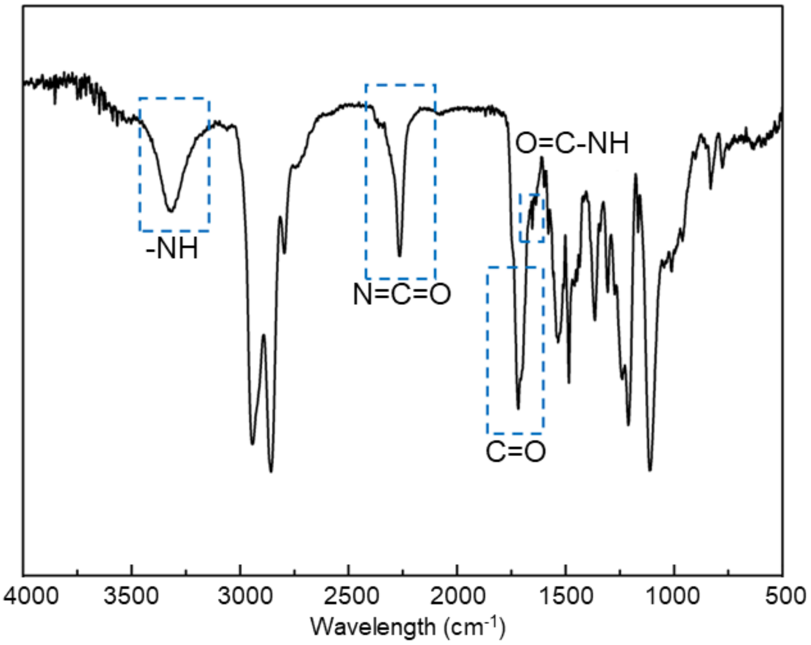

Figure S2. FTIR spectrum of the self-healable TPU.

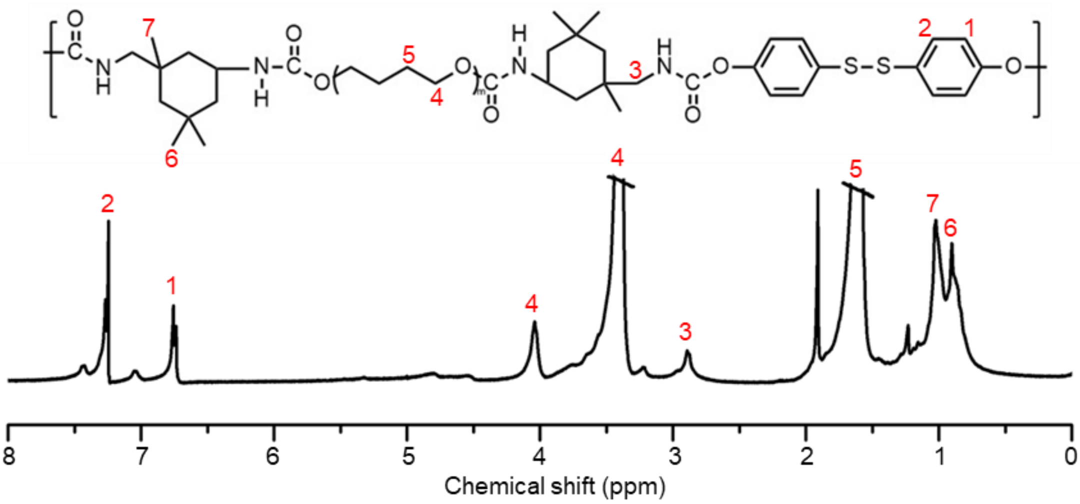

Figure S3. ^{1}H NMR of TPU.

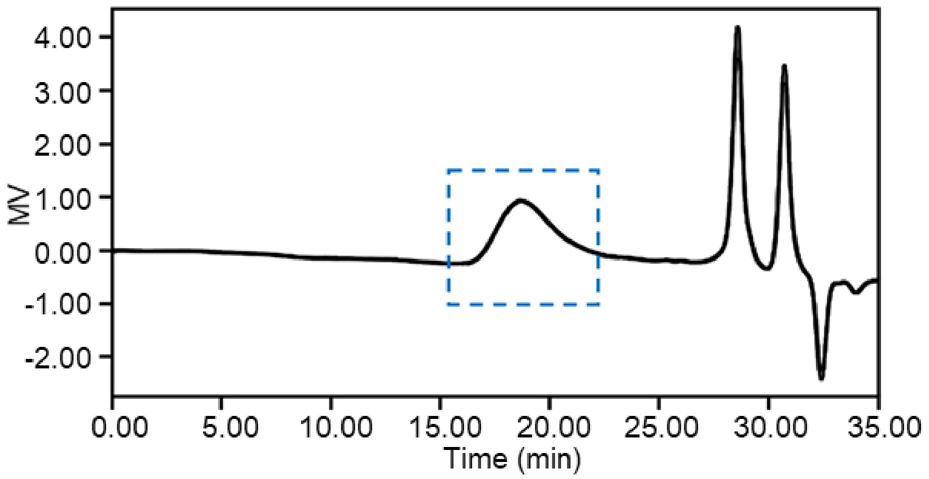

Figure S4. THF-GPC elution profile of TPU.

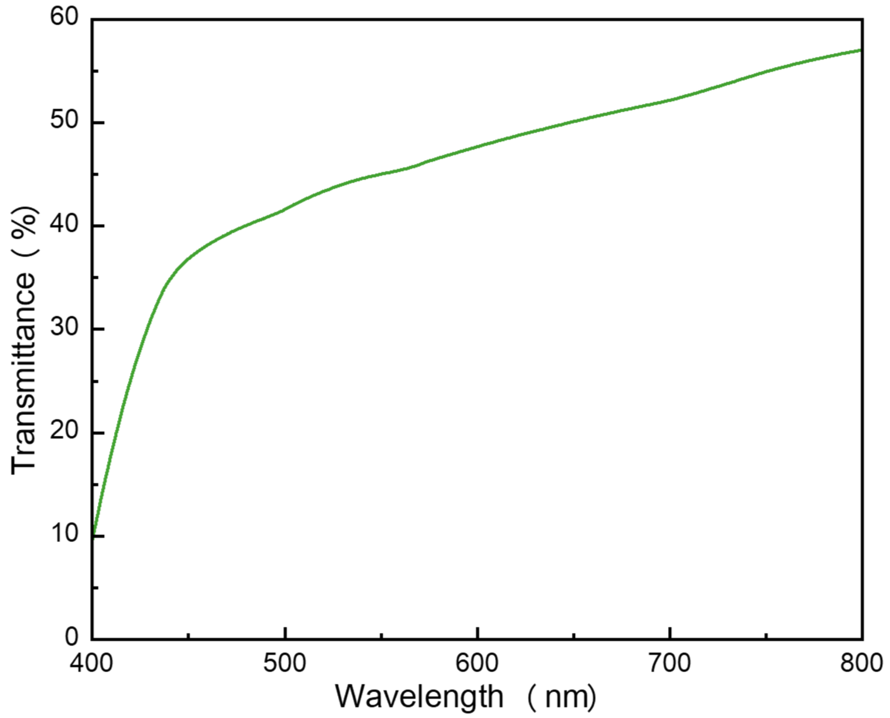

Figure S5. Transmission spectrum of the STPU film with a thickness of 120 μm.

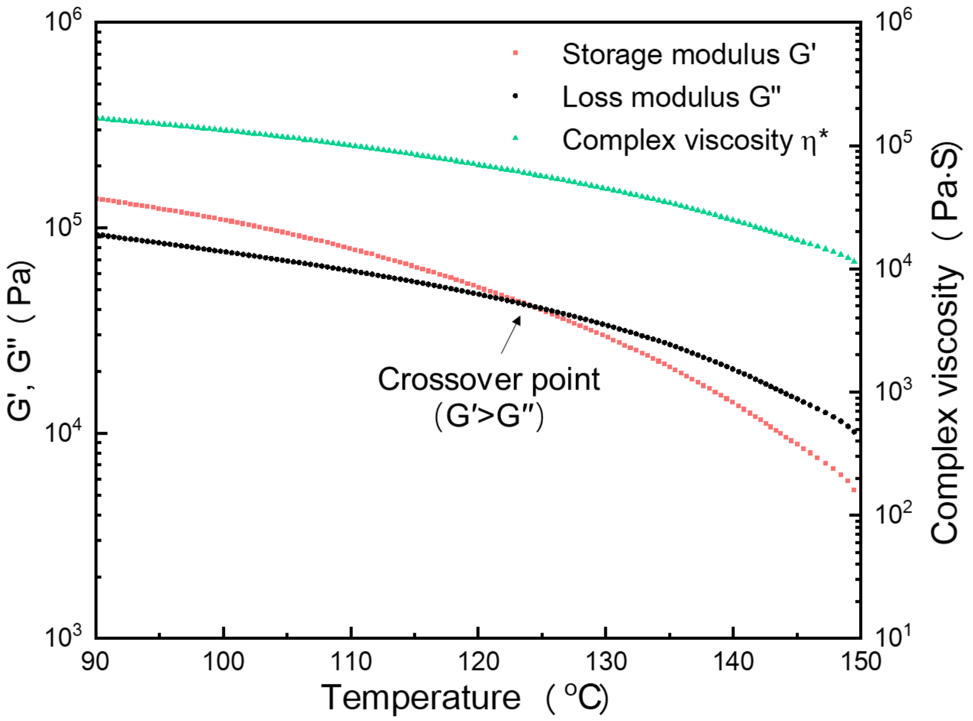

Figure S6. Rheological properties of STPU: storage modulus (G′), loss modulus (G″), and complex viscosity (η*).

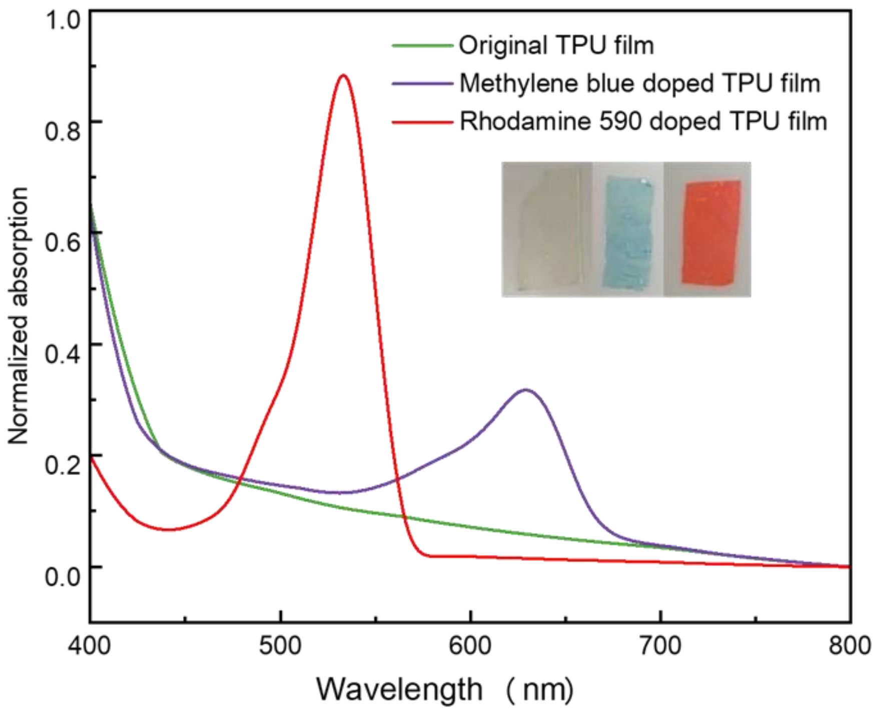

Figure S7. Absorption spectra of original TPU film, methylene blue doped TPU film, and rhodamine 590 doped TPU film. Inset: Photographs of the three TPU films.

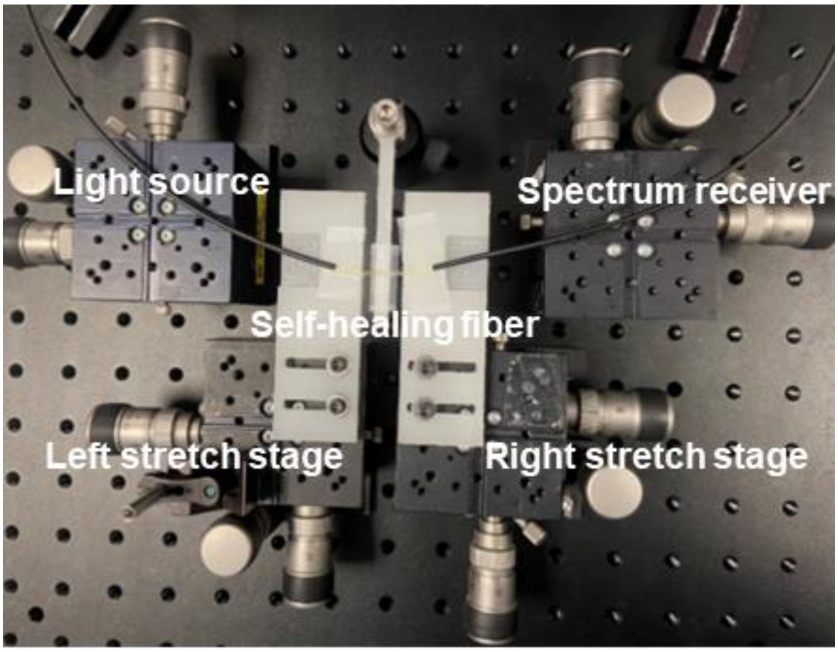

Figure S8. Optical setup for strain sensing test.

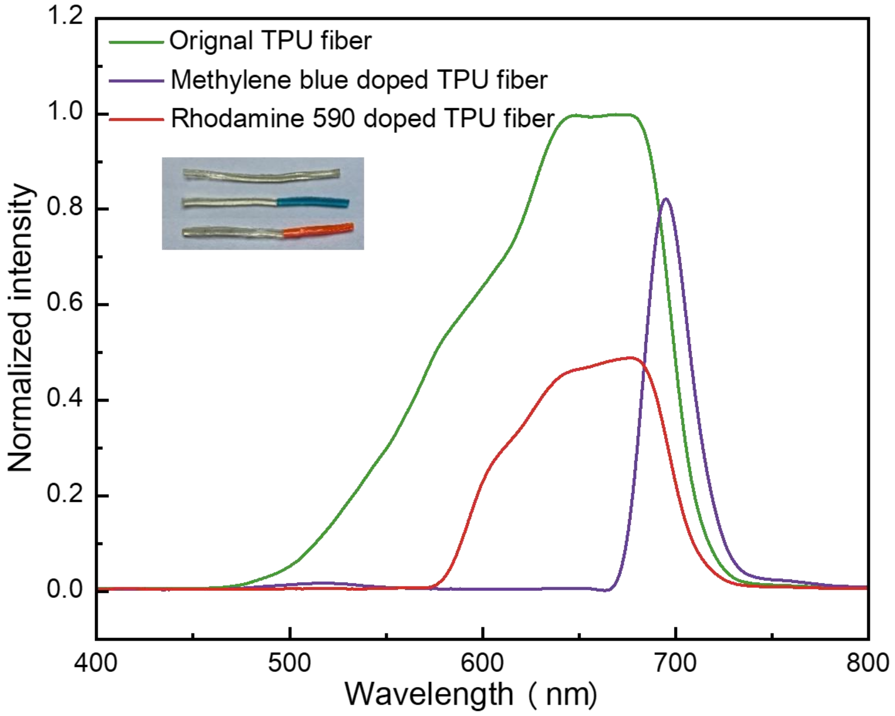

Figure S9. Normalized transmission spectra of original TPU fiber, methylene blue doped TPU fiber, and rhodamine 590 doped TPU fiber. Inset: Photographs of the three TPU fibers.

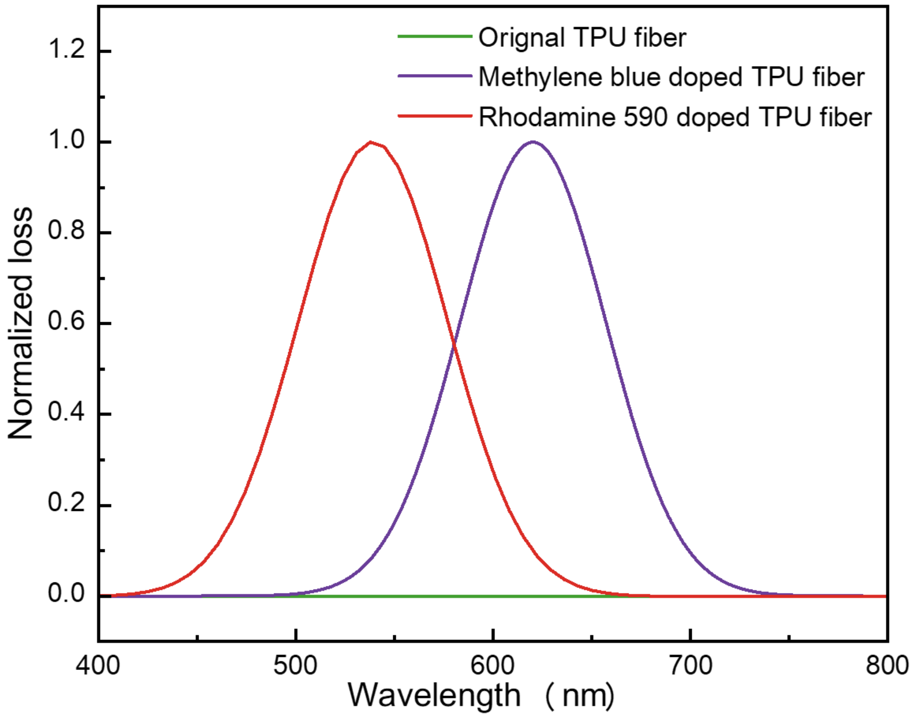

Figure S10. Normalized loss of original TPU fiber, methylene blue doped TPU fiber, and rhodamine 590 doped TPU fiber.

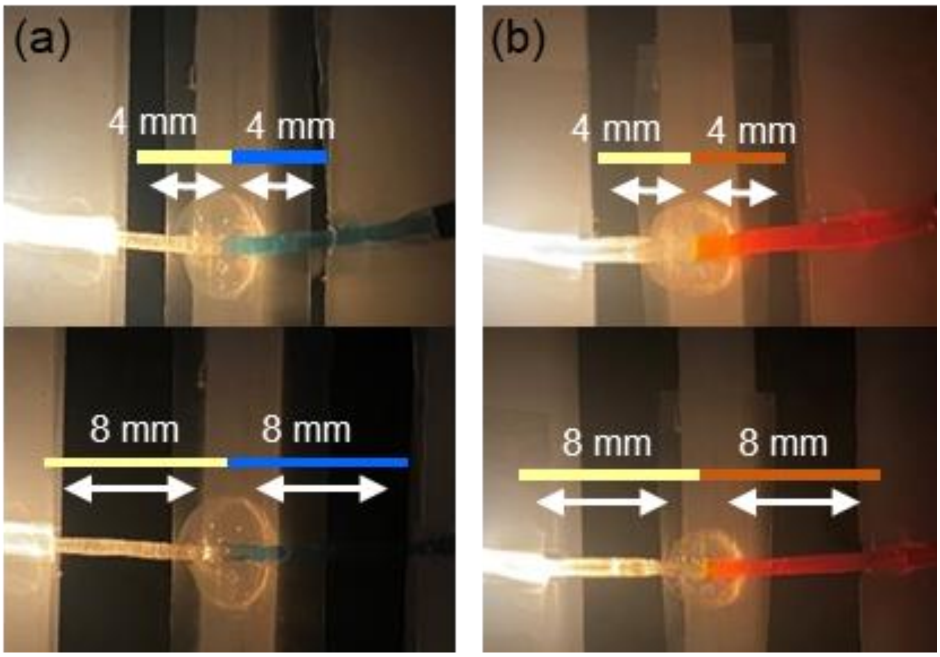

Figure S11. Pictures of methylene blue (a) and rhodamine 590 (b) doped strain sensors before and after stretching.

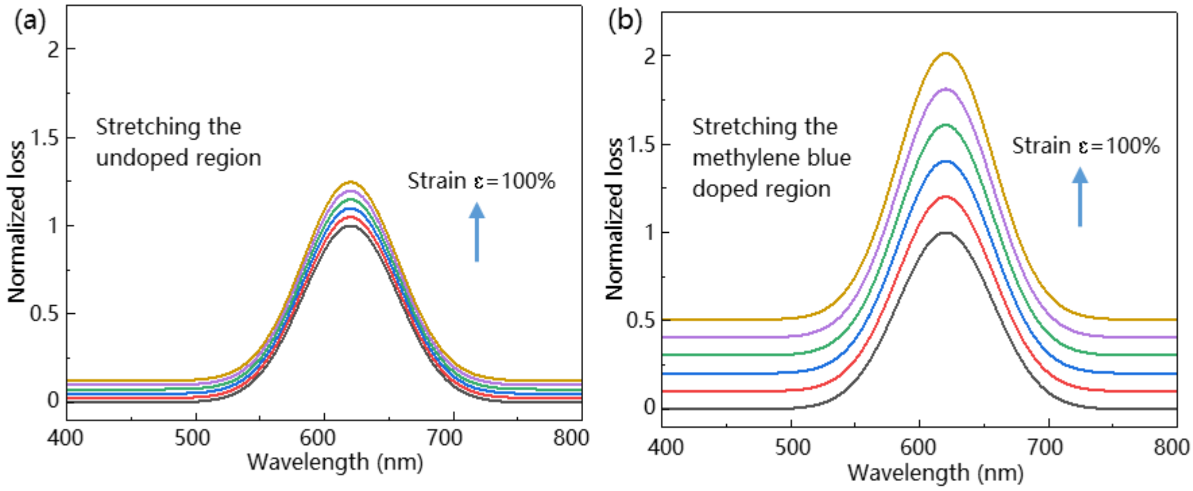

Figure S12. Normalized absorption spectra variation of the fabricated distributed strain sensor when the strain is applied to the undoped region (a) and methylene blue doped sensor region (b).

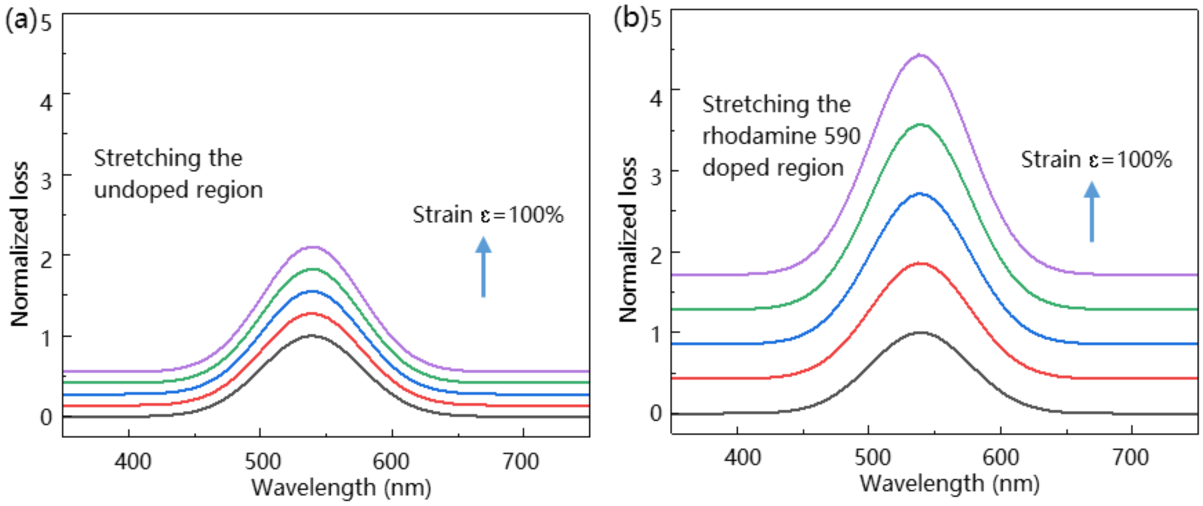

Figure S13. Normalized absorption spectra variation of the fabricated distributed strain sensor when the strain is applied to the undoped region (a) and rhodamine 590 doped sensor region (b).

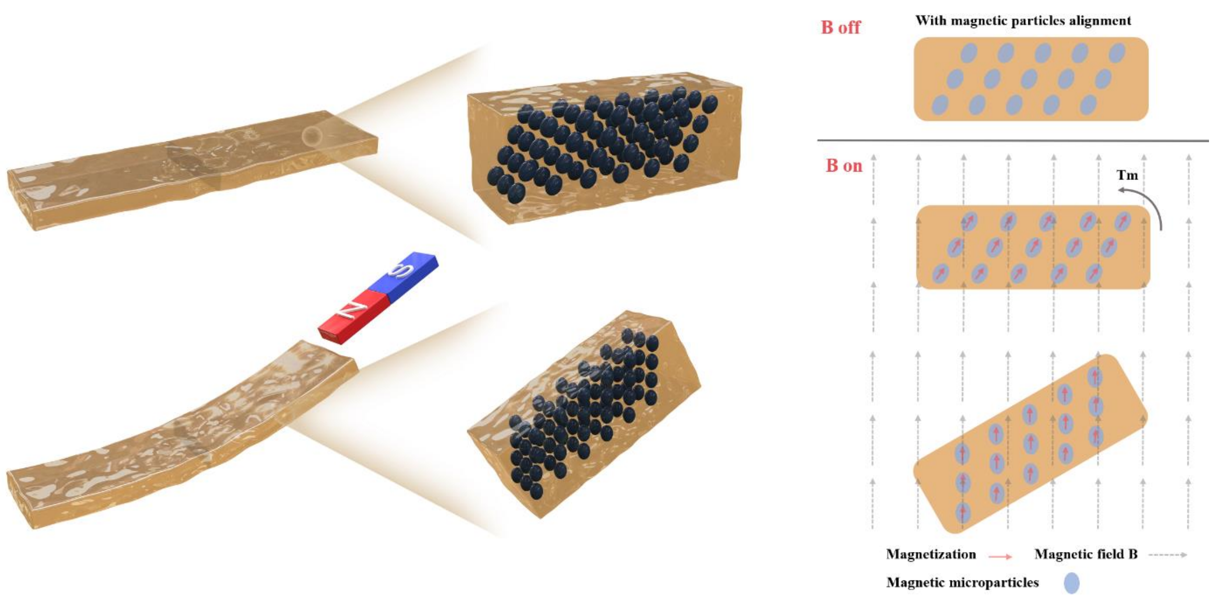

Figure S14. The mechanism of self-healing magnetic soft robot driven by magnetic torque. Magnetic actuation in self-healable TPU soft actuators is driven by magnetic torque $T_m = \mu \times M$, where B is the magnetic flux density and μ is the magnetic moment of the composite. The misalignment between an applied magnetic field and the composite material's magnetization induces torque. Embedded magnetic microparticles within the soft matrix deform until the magnetization of each domain is collinear with the applied magnetic field. This principle forms the basis for controlling the shape change of soft actuators.

References

[1] S. M. Kim, H. Jeon, S. H. Shin, S. A. Park, J. Jegal, S. Y. Hwang, D. X. Oh, J. Park, *Adv. Mater.* **2018**, 30, 1705145.
